# Role of CFTR in epithelial physiology

**DOI:** 10.1007/s00018-016-2391-y

**Published:** 2016-10-06

**Authors:** Vinciane Saint-Criq, Michael A. Gray

**Affiliations:** Epithelial Research Group, Institute for Cell and Molecular Biosciences, University Medical School, Newcastle University, Framlington Place, Newcastle upon Tyne, NE2 4HH UK

**Keywords:** CFTR, Physiology, Epithelial transport, Chloride, Bicarbonate

## Abstract

Salt and fluid absorption and secretion are two processes that are fundamental to epithelial function and whole body fluid homeostasis, and as such are tightly regulated in epithelial tissues. The CFTR anion channel plays a major role in regulating both secretion and absorption in a diverse range of epithelial tissues, including the airways, the GI and reproductive tracts, sweat and salivary glands. It is not surprising then that defects in CFTR function are linked to disease, including life-threatening secretory diarrhoeas, such as cholera, as well as the inherited disease, cystic fibrosis (CF), one of the most common life-limiting genetic diseases in Caucasian populations. More recently, CFTR dysfunction has also been implicated in the pathogenesis of acute pancreatitis, chronic obstructive pulmonary disease (COPD), and the hyper-responsiveness in asthma, underscoring its fundamental role in whole body health and disease. CFTR regulates many mechanisms in epithelial physiology, such as maintaining epithelial surface hydration and regulating luminal pH. Indeed, recent studies have identified luminal pH as an important arbiter of epithelial barrier function and innate defence, particularly in the airways and GI tract. In this chapter, we will illustrate the different operational roles of CFTR in epithelial function by describing its characteristics in three different tissues: the airways, the pancreas, and the sweat gland.

## Introduction

### The basic toolkit for epithelial electrolyte and fluid absorption and secretion

**Fig. 1:**
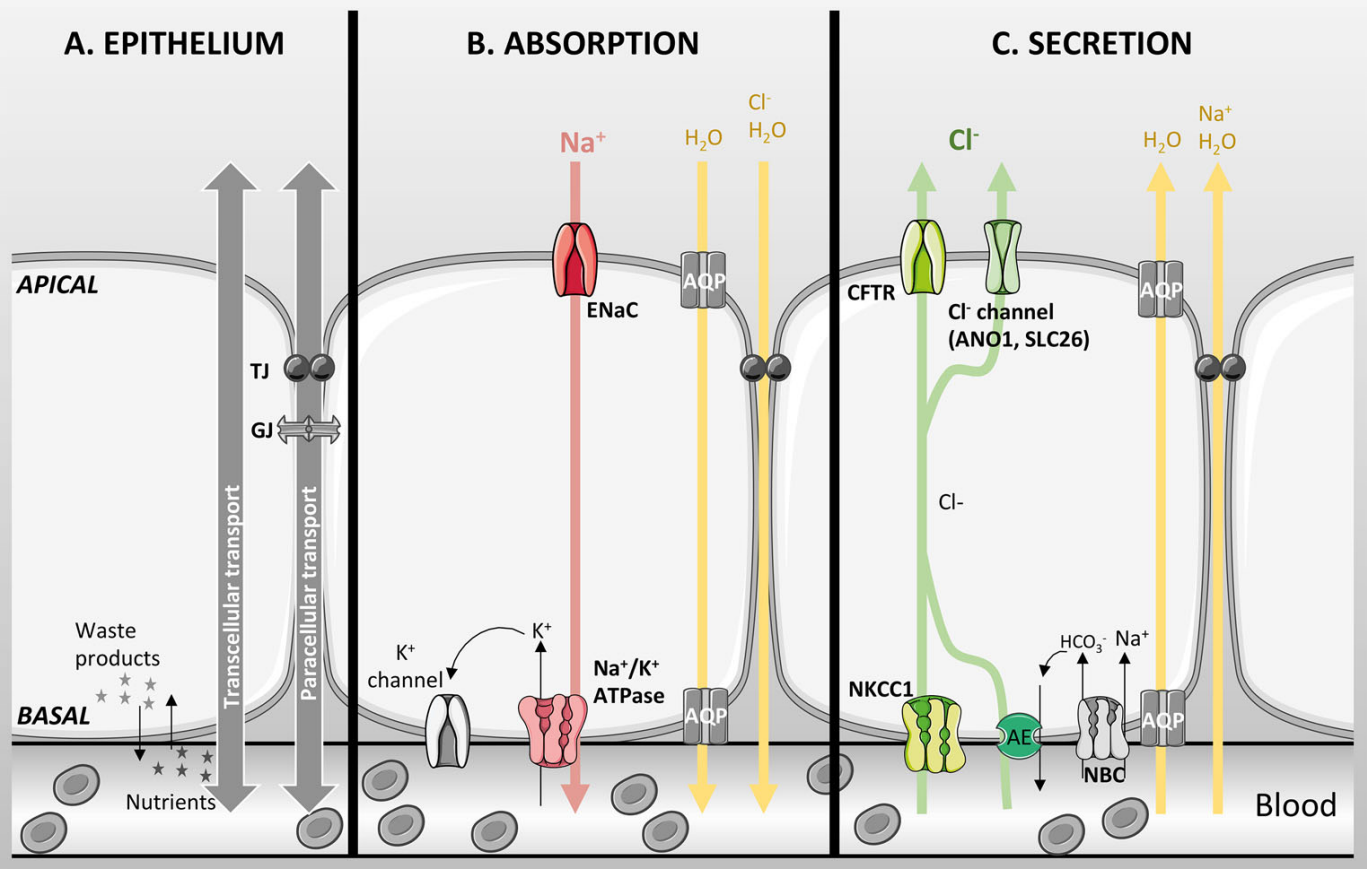
Basic characteristics of an epithelial layer. **a** Epithelial cells are joined together by junctions [tight junctions (TJ), gap junctions (GJ)]. Uptake of nutrients and oxygen, and removal of cellular waste products occur on the basolateral surface. Water and ion transport can occur through the transcellular or paracellular pathways. **b** Absorption is mainly driven by active Na^+^ absorption through ENaC in the apical membrane and the Na^+^/K^+^-ATPase in the basolateral membrane creating an electrochemical driving force for paracellular passive Cl^−^ transport. Water then follows either through aquaporins or the paracellular pathway. **c** Secretion is mainly driven by Cl^−^ secretion through CFTR and other Cl^−^ channels in the apical membrane. NKCC1 and the coupled action of an anion exchanger and NBC in the basolateral membrane accumulate Cl^−^ in the cell. Active Cl^−^ secretion creates the driving force for Na^+^ movement across the epithelium through the paracellular pathway and water transport occurs paracellularly and/or transcellularly. *Red* and *green arrows* show active transport, and *yellow arrows* show passive transport

Epithelial tissues are composed of one or more layers of closely assembled cells that cover a surface or that line a cavity. The main characteristic of epithelial cells is that they are polarised; the plasma membrane in contact with the external environment is called the mucosal or apical membrane, whereas the basolateral (serosal) membrane faces toward the interstitium. Both membranes have distinct roles due to their localization and differential expression of proteins (Fig. 1a). The basolateral membrane uptakes nutrients, ions and oxygen from the blood, and disposes of the cellular waste products. Because the apical membrane is in contact with the external environment, it serves both as a physical and chemical barrier to prevent potential pathogens or toxic matter reaching the bloodstream. The epithelial cells are joined together by points of contacts separating the apical and basolateral membrane: the interstitium is separated from the apical external milieu by junctional proteins. These proteins were originally described by histologists and grouped into four different structures: the zona occludens (tight junctions), zona adherens, macula adherens, and gap junctions. These are fundamental to maintain the polarity of the epithelia and, therefore, for the directional movement of ions and fluid that underscore the processes of absorption and secretion [1]. Gap junctions also allow electrical communication between cells as well as the diffusion of low molecular weight solutes.

The overall mechanism for electrolyte and fluid transport depends on the tissue and mainly on the differential expression, and distribution, of ion channels, exchangers, cotransporters, and pumps. Salt and fluid move either through the paracellular (between the cells) or transcellular (through the cells) pathways (Fig. 1a). The transcellular route of electrolyte transport requires active (ATP-dependent) or passive (following electrochemical gradients) transport of ions. The paracellular route is a passive process that is ultimately controlled by the prevailing transepithelial electrochemical gradients, as well as the cation-to-anion selectivity of the tight junctions. Indeed, the differential expression of claudins, which have been extensively studied in kidney epithelia [2–4], have been shown to regulate paracellular permeability, through their ability to modulate the charge, water, and size selectivity of the tight junctions [5]. Furthermore, recent patch clamp studies from polarised cultures of kidney epithelial cells have shown that claudin-4 appears to act as a chloride-selective pore, with properties very similar to conventional membrane spanning ion channels [6] Transepithelial electrical resistance (TEER) measurement is used to assess the barrier function of epithelial tissues, or cells grown on semi-permeable supports, and reflects the paracellular as well as transcellular conductivity. It is known that specific tight junction proteins largely influence epithelial resistance [7], and in the early 1970s, Frömter and Diamond used this measurement to classify epithelia into leaky (with a low TEER) and tight (TEER > 500 Ω cm^−2^). Leaky epithelia also generate a small transepithelial voltage (Vt) that is due to a low junctional resistance as well as to electroneutral transport (i.e., cotransport of a cation with an anion or exchange of an anion for another anion). In contrast, tight epithelia can produce high Vt, and are able to maintain large ion concentration and osmotic gradients [8]. Therefore, leaky epithelia are mainly involved in isotonic electrolyte and fluid transport, whereas tight epithelia are mainly involved in hypo- or hypertonic salt and fluid absorption or secretion.

Generally, salt absorption depends on the active transepithelial absorption of sodium (Na^+^) ions, which creates an electrochemical driving force for passive chloride (Cl^−^) transport in the luminal to basolateral direction (Fig. 1b). This, in term, creates a salt concentration gradient across the epithelium provoking water to be absorbed passively by osmosis. Na^+^ absorption is generally governed by the activity of the apically located epithelial Na^+^ channel (ENaC), a heterotrimer composed of alpha, beta, and gamma subunits. Na^+^ efflux across the basolateral membrane is then driven by the Na^+^/K^+^-ATPase, which ultimately maintains an inwardly directed Na^+^ gradient necessary for absorption. In addition, both apical and basolateral K^+^ channels are essential to maintain a suitable negative membrane potential for Na^+^ influx to occur (Fig. 1b).

In contrast, transcellular salt secretion is mainly controlled by Cl^−^ exit across the apical membrane and this occurs predominantly via CFTR, although a number of distinct Cl^−^ channels, such as calcium-activated Cl^−^ channels (CaCC), and solute carrier (SLC)26A9, are also involved, depending on tissue. Secretion depends on active Cl^−^ transport, which creates the driving force for Na^+^ movement across the epithelium through the paracellular pathway (Fig. 1c). The increased salt concentration on the luminal surface generates an osmotic driving force for water to be secreted, producing an isotonic secretion. For both processes, water moves either passively through the paracellular pathway or transcellularly via aquaporins, or by both routes [9–11] (Fig. 1b, c). It is important to note that in addition to Cl^−^, CFTR and ANO1 also conduct HCO_3_^−^ and that transport of this anion will also contribute to transepithelial fluid secretion (not shown on Fig. 1). Under normal physiological electrochemical gradients, and because CFTR is about 5 times more conductive for Cl^−^ than HCO_3_^−^, transport of Cl^−^ by CFTR accounts for the majority of fluid secretion in most secretory tissues. However, the secretion of HCO_3_^-^ does play a key role in modulating the pH of the secreted fluid, which is discussed in more detail below.

In CFTR expressing tissues, fluid secretion is primarily controlled by the extent of transcellular Cl^−^ transport, with the rate of Cl^−^ exit across the apical membrane being the rate-limiting step. Chloride secretion is essentially a two-stage process, which begins with the active accumulation of Cl^−^ across the basolateral membrane, through the Na^+^ K^+^ 2Cl^−^ cotransporter (NKCC1), a secondary-active transporter that uses the inwardly directed Na^+^ gradient, established by the Na^+^/K^+^-ATPase, to accumulate Cl^−^ above electrochemical equilibrium. In addition, recent evidence suggests that a basolateral Cl^−^/HCO_3_^-^ anion exchanger (most likely SLC4A2) working in parallel with an Na^+^-bicarbonate cotransporter (NBC) can also accumulate Cl^−^ within some epithelial cells [12]. In many epithelial tissues, Cl^−^ exit across the luminal membrane occurs via CFTR. The total CFTR Cl^−^ conductance of the apical membrane is dependent on three parameters: the activity, or open state probability (Po) of CFTR, which is controlled predominantly via cAMP/PKA phosphorylation as described in chapter “Biochemistry and physiology of CFTR”; the number or density of CFTR channels (N), and finally the single channel conductance (*G*_s_), which itself is governed by the electrochemical gradient across the apical membrane (membrane potential and Cl^−^ concentration). The net transport of Cl^−^ across the epithelium then drives passive Na^+^ transport via a cation-selective paracellular route, and water follows osmotically. Importantly, as Cl^−^ exits the cells the apical membrane will depolarise which will ultimately limit Cl^−^ secretion, as the apical membrane potential moves towards the Cl^−^ equilibrium potential. In some epithelial cells, intracellular Cl^−^ also falls due to CFTR activity, which can lead to activation of several protein kinases that alter CFTR anion permeability (see section on pancreas).

The role of CFTR in epithelia has been extensively studied in relation to the affected organs in cystic fibrosis (CF). As stated in Chapter “Cystic Fibrosis: a clinical view”, CF is the most common genetically inherited disease in Caucasian populations (1 in 3500 newborns in Europe) [13, 14] and 70–90 % of CF individuals harbour the F508del mutation on at least one allele [15], which results in misfolding and incorrect processing of CFTR to the apical membrane. One of the first symptoms associated with CF, and occurring in around 17 % of CF patients, is meconium ileus; an obstruction of the bowel due to thick meconium and 98 % of babies with meconium ileus has CF. Distal intestinal obstruction syndrome also has a lifetime prevalence of 8 % in children with CF and 16 % in adult CF patients, and constipation occurs in around 50 % of CF patients [16] (median of 16 % in the general population [17]). Moreover, historically, some of the first suspected descriptions of CF come from the XVI century and associated a salty skin and pancreatic damage with early childhood death [18]. Finally, the main cause of mortality and morbidity in CF is the lung pathology in which thick and sticky mucus blocks the airways promoting the development and persistence of virulent pathogens. To develop further the fundamental, but contrasting, roles of CFTR in the physiology of epithelia, we will describe its role in three different CF-affected organs; the lungs, the pancreas, and the sweat gland. However, it is important to note that CFTR plays a fundamental role in electrolyte transport throughout most of the intestinal tract, as well as in the female and male reproductive tracts, but due to space limitations, these topics will not be discussed further. For the interested reader, several recent reviews describe in detail the role of CFTR in anion (Cl^−^ and HCO_3_^−^) and fluid transport in these tissues, together with diseases associated with defective CFTR, such as CF secretory diarrhoeas and infertility [19–24].

## Role of CFTR in the lungs

**Fig. 2:**
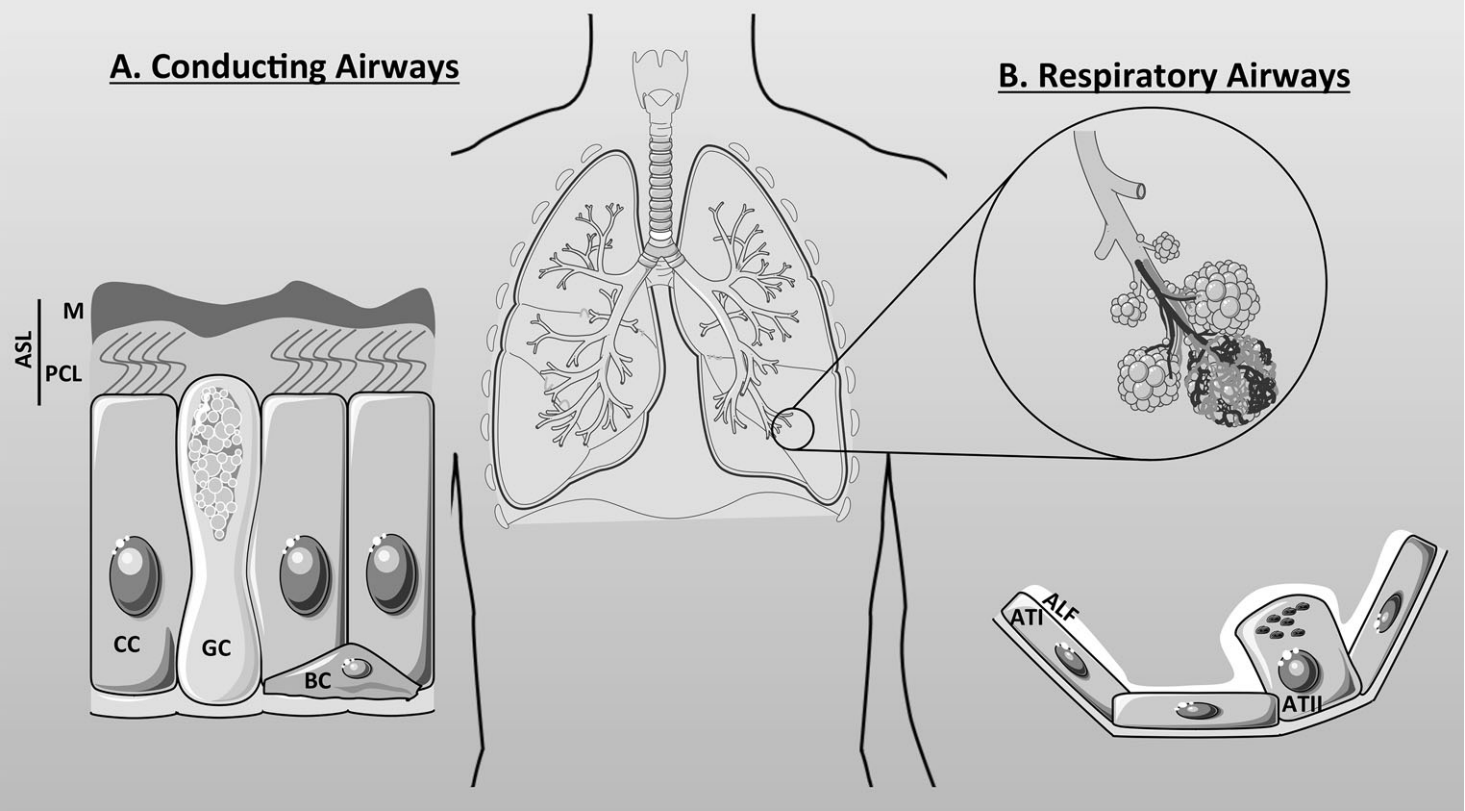
Cellular components of the airways. The airways are composed of the conducting airways (**a**) and the respiratory airways (**b**). **a** The conducting airways are covered by an aqueous film called the airway surface liquid (ASL) which is composed of the periciliary layer (PCL) and the mucus layer (M). Three types of cells constitute the conducting airways: the ciliated cells (CC), goblet cells (GC), and basal cells (BC). **b** The respiratory airways are composed of the alveolar type I (ATI) and type II (ATII) cells and are covered by the alveolar lining fluid (ALF) that prevent alveoli from collapsing

The main function of the lung is to provide oxygen to the bloodstream and, therefore, to all organs in the body, and to remove carbon dioxide from the bloodstream out to the atmosphere. The lung is composed of the conducting airways and the respiratory airways (Fig. 2). Both entities serve a particular function. The conducting airways composed of the nose, mouth, pharynx, larynx, trachea, bronchi and conducting bronchioles, conduct and warm up the air from the upper airways to the respiratory airways (respiratory bronchioles, alveolar ducts, alveoli) where gas exchange occurs. The epithelia lining the surfaces of the conducting and the respiratory airways have different cell composition: the bronchial epithelium is mainly composed of ciliated cells, goblet cells and basal cells (Fig. 2a), whereas the alveolar epithelium consists of alveolar type I and alveolar type II cells (Fig. 2b). It is also important to note that submucosal glands are found in the cartilaginous conducting airways and are composed of distinct regions with different types of cells, including ciliated and non-ciliated serous and mucous cells [25], with different functions (not shown in the figure but described below).

The conducting airways also have a role of preventing any noxious matter from reaching the alveolar gas exchange site. To accomplish this role, the epithelial cells lining the airways are protected from the inspired air by a thin (~10 μm) liquid layer called the airway surface liquid (ASL) that is composed of a lower periciliary layer (~7 μm), in which cilia beat to remove the inhaled particles and pathogens trapped in the upper mucus layer (Fig. 2a). To maintain an efficient mucociliary clearance, the process that allows the removal of trapped particulate matter by the coordination of fluid secretion and ciliary beating, ASL hydration is tightly regulated by the transport of ions and water across the surface epithelia as well as by fluid secretion arising from submucosal glands. Indeed, Ballard et al. provided evidence that the ASL is mainly secreted by the glands lying below the mucosal layer. They showed in pig airways that the rate of fluid secretion induced by acetylcholine was unaffected by the removal of the surface epithelium [26]. In submucosal glands, mucous cells are mainly localised in the tubules distal from the collecting duct, whereas the CFTR-expressing serous cells are predominantly present in the distal ducts and acini. Mucous cells secrete gel-forming mucins (mainly MUC5B) as well as fluid, but the serous cells are the primary origin of the fluid component of the gland secretions, secreting solutes, fluid and innate defence molecules [27]. Although, in the upper airways, much of the ASL originates from the submucosal glands, surface epithelia have a critical role in regulating final ASL volume and composition. This is because surface airway epithelial cells express both ENaC and CFTR and, therefore, are able to absorb Na^+^ as well as secrete Cl^−^. Studies using thin film cultured human bronchial epithelial cells (HBEs) have shown that both Na^+^ absorption and Cl^−^ secretion regulate ASL height and composition [28, 29]. In CF airways, the ASL is depleted which strongly affects mucociliary clearance and, therefore, the eradication of bacterial infections. Moreover, it was shown by Joo et al. that cAMP agonists failed to induce secretion by submucosal glands from CF patients [30]. As stated above, because of the importance of the submucosal glands in the formation of the ASL, it is likely that any defect in CFTR in these glands will strongly affect ASL homeostasis. Although CFTR is apparently not expressed in mucous cells [31], the defect in fluid secretion from serous cells affects mucin secretion (by mucous cells) as well as its hydration [32] and leads to severe mucus plugging within the glands, probably because of an acidic environment within the glands (see below).

In surface epithelia, ASL depletion was thought to be due mainly to a lack of Cl^−^ secretion that leads to a lack of an osmotic gradient, which, in turn, prevents fluid secretion, but it may also involve enhanced absorption through ENaC. Indeed, it has been shown that CFTR regulates other ion channels and transporters, and critically is involved in ASL pH regulation and innate immunity through regulation of secretion of antioxidant and antimicrobial molecules. All these functions of CFTR act together to maintain a close to sterile environment in the lungs that prevents pathogens and noxious agents from entering the blood circulation. Indeed, proper hydration of the ASL is crucial for an efficient mucociliary clearance, and recent studies have shown that ASL pH homeostasis is essential for bacterial killing [33, 34], mucus rheology [35] and fluid homeostasis [36].

### Regulation of airway surface fluid composition and hydration

Surface airway epithelial cells, as well as serous cells of the submucosal glands, secrete Cl^−^ and HCO_3_^−^ in response to agents increasing intracellular cAMP (VIP, adenosine and noradrenaline) and/or Ca^2+^ (acetylcholine, histamine or ATP) and this controls ASL volume and composition. As described in the introduction, for many epithelial cells, the Na^+^/K^+^-ATPase actively transports Na^+^ out of the cells producing a transmembrane electrochemical gradient allowing for the cotransport of Na^+^, K^+^, and Cl^−^ through NKCC1 at the basolateral membrane, thereby increasing intracellular Cl^−^ concentration above equilibrium. In serous cells, it has been shown that Cl^−^ can also be accumulated at the basolateral membrane by two other mechanisms: (1) through the coupled action of a Na^+^/H^+^ exchanger (NHE) and a Cl^−^/HCO_3_^-^ exchanger and (2) parallel operation of a Cl^−^/HCO_3_^−^ exchanger with NBC [12, 37]. At the apical membrane, Cl^−^ is secreted through CFTR, CaCCs or SLC transporters and channels.

**Fig. 3:**
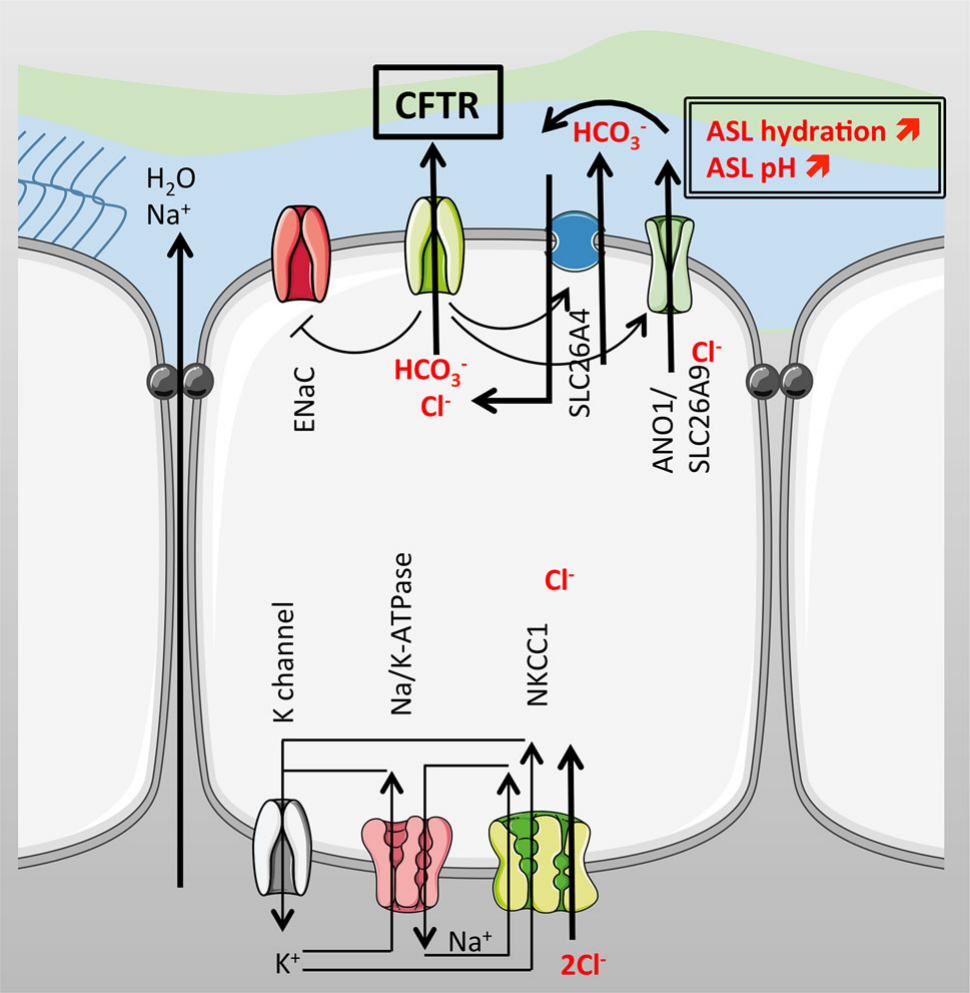
Ion transport in the airways. On the apical surface, CFTR drives Cl^−^ and HCO_3_^−^ secretion and regulates Na^+^ absorption by inhibiting ENaC. CFTR also positively regulates the Cl^−^ channels ANO1 and SLC26A9 as well as the anion exchanger SLC26A4, increasing Cl^−^ and HCO_3_^−^ secretion and therefore increasing ASL hydration and pH. Na^+^ and water follow the electrochemical gradient through the paracellular pathway. On the basolateral membrane, NKCC1 accumulates Cl^−^ intracellularly supported by the Na^+^/K^+^-ATPase. K^+^ recycling across the basolateral membrane occurs for proper function of the Na^+^/K^+^-pump

In CF, an enhanced Na^+^ absorption has also been reported and one hypothesis to explain this finding is that CFTR normally downregulates ENaC activity in non-CF cells. This was first suggested from studies in which measurements of nasal potentials in CF patients showed a large response to amiloride (a highly selective ENaC inhibitor) that was not seen in non-CF patients [38, 39]. Later work from a β-ENaC overexpressing mouse [40] provided further support, since these transgenic mice exhibited a lung phenotype characteristic of human CF airways. Indeed, airway epithelia isolated from this model showed CF ASL depletion, dehydrated mucus, neutrophilic inflammation, and poor bacterial clearance. However, the regulation of ENaC by CFTR is an ongoing debate as some studies do not report the same results [41]. In an attempt to reverse the β-ENaC mouse phenotype, Grubb et al. [42] developed another mouse strain overexpressing human CFTR that were bred with the β-ENaC mice. The hypothesis was that increasing the amount of CFTR would restore a “normal” CFTR/ENaC ratio and reverse the CF-like phenotype of β-ENaC mice airways. In this study, the double transgenic mice (hCFTR/β-ENaC) showed the same phenotype as β-ENaC mice, characterised by a reduced survival rate, airways obstruction and depleted ASL showing that in this model, CFTR was not able to rescue the lung phenotype observed in the β-ENaC mice [42]. There are a large number of studies supporting the regulatory effect of CFTR on ENaC in human airways (Fig. 3), but the mechanisms involved are still unclear due to contradictory results in different studies. A 2011 patch-clamp study from Lazrak et al. reported that CFTR regulates ENaC activity in isolated type II alveolar cells, even when CFTR protein levels were minimal [43]. On the other hand, the recent generation of CF pigs (both CFTR−/− and F508del) do not show any increased Na^+^ or water absorption compared to wild-type animals, in spite of an increased amiloride-sensitive voltage, and short-circuit current, observed in airway epithelial cultures from these animals [44]. Although the direct or indirect regulatory effect of CFTR on ENaC still needs to be confirmed, several groups are pursuing ENaC inhibitors as a therapeutic approach to increase ASL depth, and the available data show that interfering with ENaC activity could potentially be beneficial in CF to rehydrate the ASL [45].

To maintain an optimally hydrated ASL, CFTR also regulates other Cl^−^ channels and transporters, some of which belong to the SLC26 family [46]. The mammalian SLC26 family of anion exchangers and channels is composed of 10 genes that encode proteins with a C- and N-termini that frame a transmembrane domain. In the C-terminus, there is a regulatory region called the STAS (sulphate transporter and anti-sigma factor antagonist) domain. Mutations in human SLC26 genes lead to tissue specific diseases such as autosomal recessive non-syndromic deafness, DFNB4, and Pendred Syndrome in the SLC26A4 gene [47], and studies in mice models have shed light on the organ distribution of other SLC26 transporters. Among the 10 identified members of the SLC26 family, two have been shown to be expressed in airway cells, SLC26A4 and SLC26A9. Importantly, genome-wide association studies have recently demonstrated a significant association between single nucleotide polymorphisms (SNPs) in SLC genes and CF disease severity. Correlations were found with susceptibility to meconium ileus, *Pseudomonas* infections and decline in pulmonary function in CF children (SLC9A3; Na^+^/H^+^ exchanger) and CF-related diabetes (SLC26A9; Cl^−^/HCO_3_^−^ transporter) [48, 49]. Among the latest discovered SLC26 are the SLC26A7, A8, and A9 [50]. SLC26A9 is predominantly expressed in the brain and lungs, and recent work suggests that it plays an important role in airways hydration and mucus homeostasis as a polymorphism in the 3’UTR region has been shown to be associated with asthma [51]. To study the function of this transporter, Lohi et al. injected *Xenopus laevis* oocytes with SLC26A9 cRNA and measured the uptake of radiolabelled sulphate, Cl^−^ and oxalate. They reported the transport of all three anions by SLC26A9 and an inhibition of sulphate transport by DIDS and thiosulphate, implying that the protein acted as an anion exchanger, similar to several other SLC26 members (A3, A4 and A6). Further characterisation of this transporter reported a small effect of the specific CFTR inhibitor, CFTR-Inh172, in patch-clamp experiments from oocytes [52] but a more significant effect of GlyH-101 (another CFTR inhibitor, but with lower selectivity) in SLC26A9-transfected HEK 293 [53]. However, it was shown in 2009 that SLC26A9 was regulated by CFTR in HBEs and that the forskolin response was enhanced in cells coexpressing CFTR and SLC26A9, when compared to HEK-293 expressing CFTR alone. Since the increase in current was much greater than the addition of each response alone, this suggested that both channels interacted synergistically in response to forskolin. Moreover, combined data from patch-clamp experiments in HEK 293 cells and short-circuit current experiments in HBE cells showed that the constitutive basal Cl^−^ current observed in HBE cells was likely to be due to SLC26A9. This current was absent from HBE cells cultured at air liquid interface (ALI) from CF patients, and furthermore, these cells lacked the signature currents, typical of SLC26A9 alone or coexpressed with WT-CFTR in HEK 293 coexpressing F508del-CFTR and SLC26A9 [53], suggesting that the mutant CFTR prevented SLC26A9 activity. However, in the same year, Chang et al. showed that this anion transporter was inhibited by the regulatory domain of CFTR and that this effect occurred through an interaction with the STAS domain of SLC26A9 [54]. Clearly, the effect of CFTR on SLC26A9 activity requires further investigation, but in the airways at least, it seems both channels work together to maintain ASL hydration (Fig. 3).

CFTR has also been shown to regulate Ca^2+^ activated Cl^−^ secretion, potentially via changes in CaCCs (Fig. 3). The histamine or UTP peak secretory response was enhanced in cultured CF airway epithelial cells and freshly excised nasal tissues from CF patients, respectively [55, 56]. In CF mice, enhanced Ca^2+^-dependent Cl^−^ secretion was also detected [57]. In a study from 2011, soon after the identification of the CaCC as being encoded by TMEM16A (also called Anoctamin 1, ANO1) [58–60], Ousingsawat et al. reported that the activation of CFTR inhibited ANO1 in a bronchial epithelial cell line [61] and also showed a molecular interaction between the two channels in an overexpressing model using HEK293 cells. These are a few examples of how CFTR regulates other channels to maintain a fully hydrated ASL. However, CFTR is also involved in regulating the movement of water across the epithelium through the transcellular pathway by regulating aquaporins such as aquaporin 3 in non-CF cells [62]. Studies performed in CF and non-CF cell lines, by two different groups, also showed that CFTR modulates the paracellular pathway. When CFTR was activated in non-CF cells, the transepithelial electrical resistance decreased and paracellular conductance increased. CFTR inhibition led to a disorganisation of F-actin and α-tubulin that was also observed in CF epithelia [63] and involved a myosin II dependent mechanism [64].

Another hallmark of CF airway disease that is also found in COPD is the accumulation of mucus that forms mucus plugs on the surface of airway epithelial cells. The composition of mucus includes water, ions, and macromolecules with protective functions such as anti-microbial, anti-protease, and anti-oxidant activity. Mucins are glycoproteins that are responsible for the viscoelastic property of mucus, which is crucial for an effective mucociliary clearance. Because organs affected in CF are mainly mucus producing epithelia, it has been proposed that a defect in CFTR expression and/or activity would affect mucus production and properties [65]. However, the fact that some mucus producing organs do not show complete obstruction in CF, such as the salivary and lacrimal glands, suggest an indirect role for CFTR in the regulation of mucus production. Moreover, the phenotype of the overexpressing β-ENaC mice (described earlier in this chapter) suggested that CFTR affects mucus by activating and/or inhibiting other factors, such as pH and HCO_3_^−^.

In the lower airways, alveolar cells must remove water from their surface to permit gas exchange. This is a major function after birth when the lungs are filled with amniotic fluid, but it is also involved in the clearance of fluid in the resolution of pulmonary oedema in adulthood. Cardiogenic lung oedema is due to an elevated hydrostatic pressure secondary to a high pulmonary venous pressure. It was thought that fluid movement into the alveolar lumen was due to a passive movement of water through the paracellular pathway due to the elevated pressure. However, a recent study has shown that active signalling could be involved in this process and that CFTR could mediate alveolar Cl^−^ and fluid secretion [66]. However, other studies have shown that this anion channel is also involved in fluid resorption in alveolar cells. Na^+^ absorption plays a crucial role in this mechanism [67–70], but recent evidence points towards a control of fluid absorption by CFTR. Using in situ perfused lungs from wild-type mice, Fang et al. showed that inhibition of Cl^−^ channels, as well as Cl^−^ substitution by gluconate, inhibited fluid clearance by 50 % [71]. Increasing intracellular cAMP led to an increase in fluid clearance, and this could be inhibited by addition of glibenclamide, an inhibitor of CFTR, confirming the role of CFTR in alveolar fluid resorption. The same group validated their findings in primary human alveolar type II cells cultured at air–liquid interface. Inhibition of CFTR with CFTR-Inh172 did not have any effect on basal fluid absorption but inhibited ~35 % of cAMP-induced fluid absorption [72]. A more recent study by Korbmacher et al., in which they used a novel Deuterium oxide (D_2_O) dilution method in conjunction with short-circuit measurements in Ussing chamber experiments, showed that inhibition of ENaC abolished more than 70 % of water resorption. Inhibition of CFTR (using CFTR-Inh172 or glibenclamide) reduced basal water resorption by ~15–20 %, whereas a more general inhibition of Cl^−^ channels by NPPB blocked almost 60 % of this process, demonstrating the involvement of CFTR and other apical Cl^−^ channels in water resorption across the alveolar epithelium [73].

### Regulation of the ASL pH by CFTR

Many recent studies have highlighted the importance of ASL pH regulation in airways homeostasis. Indeed, it was first demonstrated in the 1970s that the pancreatic secretions in patients with CF were acidic (see section on pancreas), and some years later, it was hypothesized that the defect in HCO_3_^−^ secretion caused by defective CFTR could also occur in the lungs, and therefore decrease the ASL pH [74]. An acidic ASL pH has also been demonstrated in other lung pathologies such as asthma [75], COPD [76] and acute respiratory distress syndrome [77]. Although the link between CFTR and asthma is still controversial [78–81], it is now well established that COPD shares some common features with CF, including bronchiectasis, mucus plugging, and inflammation. The primary cause of COPD is a chronic exposure to oxidative insults such as cigarette smoking or passive exposure to cigarette smoke. Cigarette smoke exposure leads to an increase in inflammation and a decrease in CFTR activity. It was first reported in the early 1980s, before the identification of the CFTR gene, that cigarette smoke decreased Cl^−^ secretion which could not be prevented by administration of antioxidants [82]. In 2006, Cantin et al. showed that this effect was due to an inhibition of CFTR [83], and it was later demonstrated that cigarette smoke actually induced CFTR internalisation, and this led to ASL dehydration [84], potentially contributing to the development of COPD pathology.

There is now more evidence to show that the lack of HCO_3_^−^ secretion in CF airways leads to a reduced ASL pH, and this further deteriorates the lung pathophysiology. An acidic ASL pH was demonstrated in the early 2000s in primary HBE cell cultures from CF and non-CF patients [85]. This study also showed that activating CFTR by increasing intracellular cAMP, increased ASL pH in non-CF epithelia but had the opposite effect in CF monolayers. Because normal submucosal glands are capable of secreting HCO_3_^−^ and CFTR conducts this anion, it has be hypothesized that submucosal gland fluid, and therefore ASL pH, would be affected in CF. Indeed, Song et al. showed that freshly collected fluid secreted by submucosal glands from CF patients was more acidic that glands from non-CF individuals [86]. Also in a 2012 study on newborn CF pigs, Pezzulo et al. showed that the ASL was more acidic when compared to wild-type piglets. Although the antimicrobial composition of the ASL was similar in CF and non-CF piglets, the airways from the CF pigs showed a reduced efficiency in bacterial killing, and they suggested that the acidic pH was the cause for the lack of activity of antimicrobial molecules [34]. Moreover, in primary HBEs, the decreased ASL pH also increased Na^+^ and fluid absorption, because the secreted protein short palate lung and nasal epithelial clone 1 (SPLUNC1) effectively was not able to inhibit ENaC-dependent Na^+^ absorption and preserve ASL volume, when ASL pH was acidic [36]. Finally, mucus properties are also regulated by pH, and different groups have shown the negative impact of an acidic pH on mucus rheology [65, 87–89]. In a review published in 2008, Quinton hypothesized that the original cause of the different organ pathologies in CF would be the loss of HCO_3_^-^, which would affect mucin expansion and mucus gel formation. Indeed, before they are released, mucins are in a very condensed form in granules. This compact form is maintained within the granules by a high concentration of cations, specifically Ca^2+^ and H^+^ [90, 91]. Upon release of mucins, HCO_3_^-^ would complex with Ca^2+^ and H^+^, allowing mucin chains to fully expand and form a gel [65].

As stated above, CFTR regulates extracellular (luminal) pH. It appears to do this by its ability to conduct HCO_3_^-^ as well as Cl^−^. However, as discussed in more detail in the section on the pancreas, CFTR may also be able to regulate ASL pH via an indirect route through regulating the activity of SLC26 Cl^−^/HCO_3_^−^ exchangers, such as pendrin and potentially other members of the SLC26 family (Fig. 3). The reciprocal regulatory interaction between the SLC26 and CFTR involves binding of the STAS domain to the regulatory domain of CFTR (see section on pancreas). The SLC26 transporter SLC26A4, pendrin, has been shown to be upregulated in inflammatory conditions in airway epithelia such as chronic rhinosinusitis [92, 93], upon stimulation with IL-17 [94] and in response to bacterial infections [95]. The other mechanism that involves CFTR in ASL pH regulation is its capacity to regulate Cl^−^/HCO_3_^−^ anion exchange, and more particularly, SLC26A4 in airway epithelial cells. In serous cells, an increase in cAMP triggered HCO_3_^-^ secretion through the apical membrane. Using genetically modified cell lines (Calu-3 WT, CFTR knockdown and pendrin knockdown), the authors showed that this effect was CFTR-dependent but that although fluid secretion was mainly mediated through CFTR, the cAMP-induced rise in HCO_3_^−^ secretion was mediated via pendrin [96].

### Regulation of pulmonary innate immunity

ASL pH is also a crucial factor for an efficient immune response to eradicate trapped pathogens and prevent their propagation. Indeed, it has been shown that the activity of molecules such as the protease inhibitor SPLUNC1, which regulates antimicrobial peptides (AMPs) and shows itself antimicrobial activity [97], depends on optimal ASL pH [36, 98], as does the activity of AMPs themselves [34]. On a more general level, when pathogens enter the airways, they are recognised by epithelial cells that activate inflammatory pathways to recruit immune cells, specifically neutrophils, into the airway lumen to eradicate the infection. It has been shown that CFTR regulates this process and when CFTR is absent, there is an upregulation of proinflammatory molecules. The current debate is whether the exacerbated inflammation in CF is due to the sustained infection or to the defective CFTR channel. In 2007, Verhaeghe et al. showed the overexpression of proinflammatory proteins in the airways of 24 weeks old CF foetuses [99]. Moreover, Tirouvanziam showed in 2000 and 2002 that a graft of non-infected tracheas or distal airways from CF foetuses on severe combined immunodeficiency (SCID) mice induced an increase in IL-8 and the recruitment of neutrophils [100, 101]. However, since the development of the CF pig model, it has been shown that inflammatory markers in bronchoalveolar lavages (BALs) of the lung did not differ from non-CF piglets, although the CF pigs showed a marked increased susceptibility to infection [102]. However, a recent transcriptomic study showed that although levels of inflammatory markers did not change, CF pig airways responded with a diminished host defence response after infection with *S. aureus*, when compared to non-CF pigs [103].

In addition, CFTR transports glutathione (GSH) [104] and thiocyanate (SCN^−^) [105], and both molecules have been shown to have a crucial role in the regulation of the immune response in the lungs. GSH is a sulfhydryl containing tripeptide that can bind and inactivate oxidants. Reactive oxygen species (ROS) can be produced endogenously (from inflammatory cells) or come from exogenous sources (pollution, cigarette smoke). Their endogenous production is important for the antimicrobial defence of the lungs, but a sustained increase in ROS can lead to increased inflammation and tissue damage. It is, therefore, important for the epithelium to be able to inactivate ROS once the infection has been eradicated. As a GSH transporter, it has been shown that a defective CFTR leads to a reduced concentration of GSH in the ASL [106] which will impair the redox balance in the airways. SCN^−^ is an anion that plays an important role in the antimicrobial defence of the lungs by reducing tissue-damaging species such as hydrogen peroxide (H_2_O_2_) and hypochlorite (OCl^−^) by subjecting itself to oxidation by lactoperoxidase, producing hypothiocyanite (OSCN^−^). OSCN^−^ itself has been shown to have antimicrobial activity [107] and SCN^−^ protected a lung cell line from injury caused by H_2_O_2_ and from OCl^−^ [108]. The absence of CFTR in CF and other inflammatory lung pathologies leads to an abnormal antioxidant composition of the ASL, worsening airway inflammation and damaging the epithelium. Epithelial tissue damage triggers signalling cascades that lead to wound repair to prevent potential pathogens to enter the bloodstream. In a process called epithelial restitution, the injured epithelial cells go through different de-differentiation and re-differentiation stages to proliferate, fill the “gap”, and recover their full functionality [109]. It has been shown that CFTR plays a critical role in this mechanism. Although it is not clear whether a defective CFTR decreases or enhances wound healing, many studies show that bronchial epithelial cells lacking CFTR are not able to reconstitute a fully differentiated epithelium [110–112].

In conclusion, CFTR has a fundamental role in the physiology of the airways. It is involved directly and indirectly in anion secretion that is crucial for airways hydration, ASL pH and defence homeostasis (Fig. 3). Potential therapies for CF have proven difficult to implement, possibly because CFTR is engaged in so many different functions in the airways. However, as correctors and potentiators of CFTR are being developed, another possible way to compensate for defective CFTR would be to target alternative ion channels or transporters such as the SLC26 family members (A4 or A9), or the anoctamins, short-circuiting their regulation by CFTR and rebalancing ASL homeostasis. One important advantage of this ‘alternate non-CFTR approach’ is that it would benefit all CF patients regardless of genotype, and would potentially be useful for other pathologies in which CFTR has been shown to be downregulated such as COPD.

## Role of CFTR in the exocrine pancreas

**Fig. 4:**
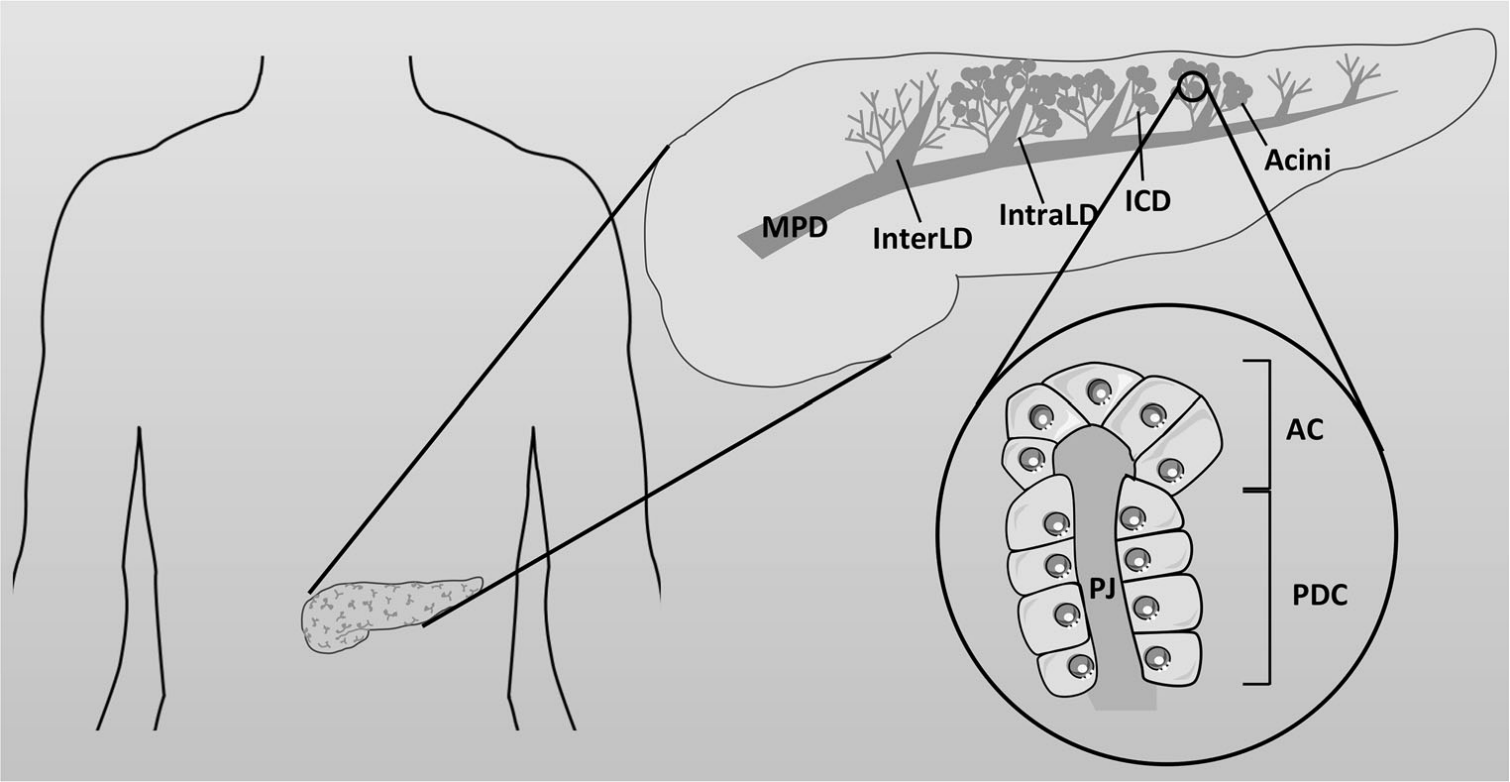
Simplified structure of the pancreas. The exocrine pancreas is composed of acini that surround a central lumen open to the duct system. Acinar cells (AC) secrete digestive enzymes into small intercalated ducts (ICD) where the pancreatic duct cells (PDC) raise the pH of the pancreatic juice (PJ). These ducts are directly connected to increasingly larger intralobular (intraLD) and interlobular (interLD) ducts that join the main pancreatic duct

The pancreas is composed of both exocrine and endocrine glands. The exocrine pancreas secretes ~2 L/day of an isotonic, HCO_3_^−^ rich fluid, containing a complex mixture of digestive enzymes (zymogens), classically known as pancreatic juice. The exocrine pancreas is composed mainly of two types of epithelial cells; acinar cells which make up ~90 % by volume of the gland, and ductal cells which make up the remaining 10 %, with a small proportion of mucus secreting and endocrine cells. The ductal cells form a complex, tubular, network which ramifies throughout the gland [113] (Fig. 4). CFTR is highly expressed in the apical membrane of ductal cells but is not present in acinar cells [114, 115], and is essential for electrolyte and fluid secretion from the gland. The importance of this fluid secretion to pancreatic function is well demonstrated in CF, where the lack of CFTR-dependent fluid secretion leads to near complete destruction of the gland at birth in ~85 % of people with CF [116, 117].

**Fig. 5:**
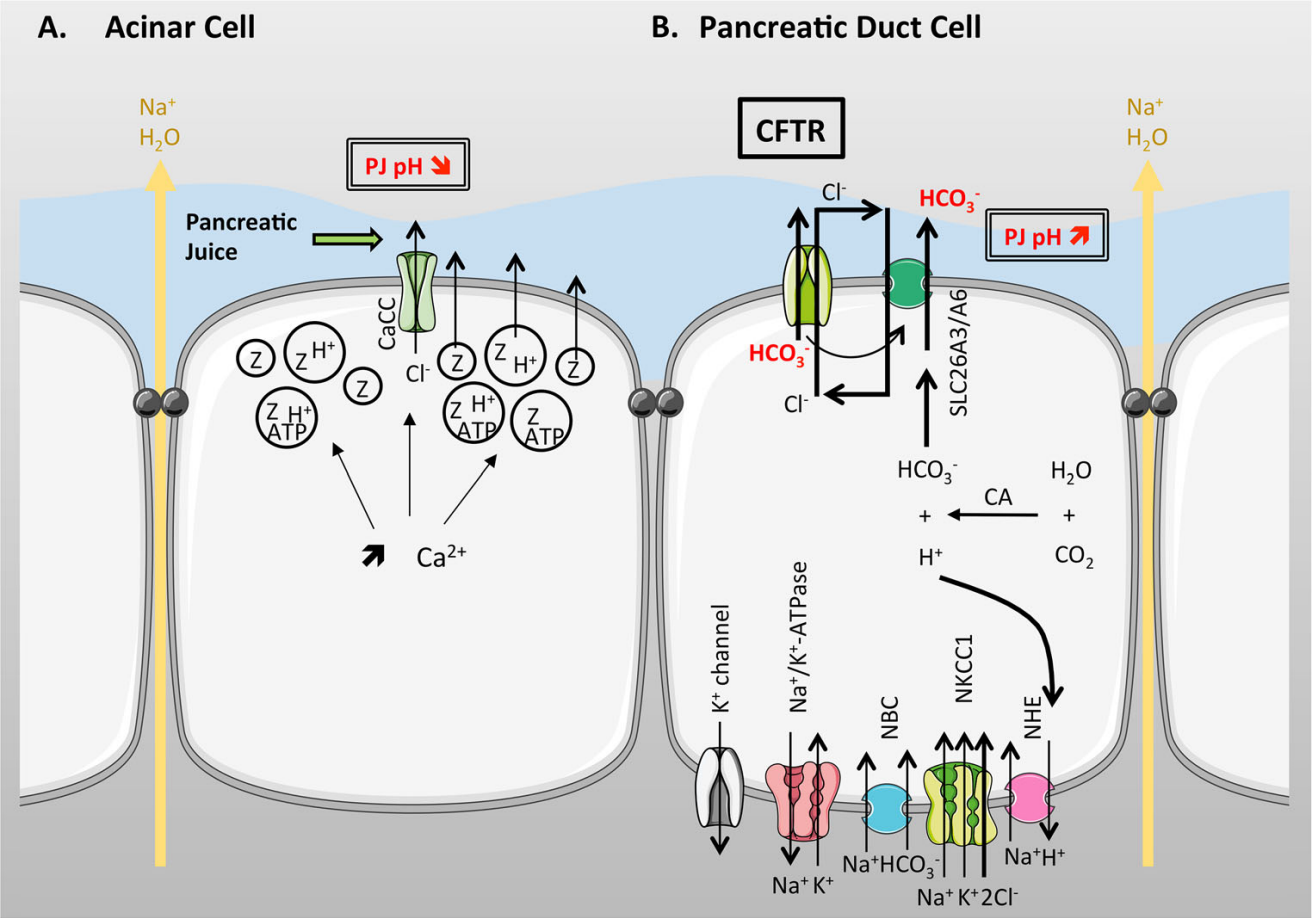
Ion transport in acinar (**a**) and pancreatic duct cells (**b**). **a** Upon stimulation with acetylcholine, cholecystokinin or other agonists, intracellular [Ca^2+^] increases and stimulates NaCl and fluid secretion, as well as the exocytosis of enzyme-containing secretory granules. As these granules also contain H^+^, the local pH falls to approximately 6.8. Cl^−^ secretion occurs through a CaCC on the apical membrane. **b** CFTR conducts Cl^−^ and HCO_3_^−^ and works in concert with a Cl^−^/HCO_3_^−^ apical exchanger, to mediate net transepithelial HCO_3_^−^ secretion, with Cl^−^ recycling across the apical membrane. Na^+^ moves paracellularly in response to transepithelial HCO_3_^−^ secretion, and water follows osmotically, to produce a HCO_3_^−^-rich isotonic fluid. Cl^−^ accumulates across the basolateral membrane via NKCC1 and accumulation of HCO_3_^−^ inside the cells occurs through the hydration of CO_2_ to HCO_3_^−^ and H^+^ by carbonic anhydrase (CA), together with backward transport of H^+^ via the basolateral Na^+^/H exchanger (NHE). This is driven by the Na^+^ gradient established by the Na^+^/K^+^-ATPase. The Na^+^-Bicarbonate cotransporter (NBC) helps accumulate HCO_3_^−^ within the cell and maintain an electrical driving force for efflux of HCO_3_^−^ across the apical membrane. It also works with K^+^ channels to maintain a negative membrane potential

In terms of functional roles, the acinar cells secrete a small volume of an NaCl-rich secretion plus many types of digestive enzymes (8–20 g per day) in an inactive form, together with various factors (e.g., ATP) that contribute to cell signalling within the ductal system [118]. Acinar cells secrete in response to stimulation by acetylcholine, cholecystokinin (CCK), and several other agonists. These agonists cause a rise in intracellular calcium which stimulates NaCl and fluid secretion, as well the regulated exocytosis of enzyme-containing secretory granules [119]. Recent studies have also shown that following regulated exocytosis the local pH falls to approximately 6.8, due to proton secretion that accompanies exocytosis [120] (Fig. 5a).

The ductal cells forming the intercalated, and/or intralobular and interlobular ducts express CFTR (varies with species) and are responsible for secreting an isotonic, highly alkaline, fluid containing up to 160 mM NaHCO_3_^−^. However, the composition of the secretion is very much flow dependent in all species studied [113, 118, 121]. There is a reciprocal relationship between HCO_3_^−^ and Cl^−^ concentrations: as fluid secretory rate increases, so does HCO_3_^−^ concentration with a corresponding reduction in Cl^−^ concentration. At maximal flow rates, HCO_3_^−^ concentration peaks around 140–160 mM in most species except the rat and mouse, where the maximum is around 70 mM [113, 118]. The major physiological regulator of pancreatic HCO_3_^−^ secretion is the peptide hormone, secretin, which is released from intestinal cells mainly in response to acid chime entering the intestine. Binding of secretin to ductal cells stimulates a rise in intracellular cAMP and activation of PKA, which leads to an increase in CFTR activity through phosphorylation of the channel, as described in detail in the chapter “Biochemistry and Physiology of CFTR”. Other transporters required for HCO_3_^−^ secretion are also regulated by cAMP/PKA (see below).

While the role of the enzymes in digestion is clear, the functions of pancreatic electrolyte secretion are less precise, but include acting as a vehicle for transporting the inactive digestive enzymes to the small intestine where the HCO_3_^-^ helps to neutralise gastric acid and so elevate duodenal pH to the optimal value required by the digestive enzymes. Since HCO_3_^−^ is a chaotropic anion, it will also aid disaggregation of secreted enzymes following exocytosis, as well as help in mucin secretion and gel formation as described in the section on the airways. In addition, HCO_3_^−^ acts to neutralise the protons that are cosecreted with enzymes from the acinar cells. Maintaining a neutral to alkaline luminal pH is important for the membrane dynamics of acinar cells and, consequently, the exocytosis of zymogen granules [122]. Furthermore, lower extracellular pH has been shown to enhance secretagogue-induced zymogen activation within acinar cells [123]. Finally, recent studies have provided strong evidence that adequate HCO_3_^−^ secretion is essential for preventing premature autoactivation of trypsin by protons within the ductal tree, and therefore inhibiting autodigestion of the gland. Indeed, trypsin itself has been shown to reduce CFTR-dependent HCO_3_^−^ secretion from duct cells [124], through activation of apical proteinase-activated receptor-2, which leads to further trypsin autoactivation. Furthermore, a fall in ductal HCO_3_^−^ secretion appears particularly important in protecting the pancreas from developing acute pancreatitis induced by stresses such as bile and alcohol [125], the two most common causes of pancreatitis in man (see below).

### Role of CFTR in pancreatic HCO_3_^−^ secretion

As described in the section on the airways, CFTR is known to conduct a variety of anions, including Cl^−^ and HCO_3_^−^. Early patch clamp studies from rat and human pancreatic duct cells demonstrated that under near physiological gradients, CFTR was approximately 5 times more permeable to Cl^−^ than to HCO_3_^−^ [126–129]. This finding, together with microelectrode studies from intact ducts that demonstrated the presence of a SITS-sensitive Cl^−^/HCO_3_^−^ exchange activity on the apical membrane together with an NPPB-sensitive Cl^−^ conductance [130, 131], combined with intracellular pH measurements [132], led to the first model of HCO_3_^−^ secretion that is shown in Fig. 5b [126, 130, 131]. In this model, CFTR was seen to act purely as Cl^−^ secretory channel that worked in concert with an apical Cl^−^/HCO_3_^-^ exchanger, to mediate net transepithelial HCO_3_^−^ secretion from the duct cells, with Cl^−^ recycling across the apical membrane. Na^+^ then moves paracellularly in response to transepithelial HCO_3_^−^ secretion, and water follows osmotically, to produce a HCO_3_^−^ -rich isotonic fluid. In essence, CFTR's role is to help maintain the activity of the exchanger by providing a source of luminal Cl^−^, as well as limiting intracellular Cl^−^ accumulation, through the operation of the exchanger, which would ultimately put a break on HCO_3_^−^ efflux by the exchanger. In this early model, Cl^−^ was shown to be accumulated across the basolateral membrane via NKCC1, and active accumulation of HCO_3_^−^ inside the duct cells was achieved through the hydration of CO_2_ to HCO_3_^−^ and H^+^ by carbonic anhydrase, together with backward transport of H^+^, via the basolateral NHE. The latter step being driven by the inwardly directed Na^+^ gradient established by the basolateral Na^+^/K^+^-ATPase. In contrast to the rat, the pig was shown to express a basolateral H^+^-ATPase that served a similar purpose to the NHE [133]. While this CFTR/anion-exchanger model for HCO_3_^−^ secretion satisfied the early electrophysiological results from the rat gland (which only secretes about 70 mM NaHCO_3_), it was soon apparent that this model had serious deficiencies and could not explain the secretion of near isotonic (140–160 mM) NaHCO_3_ juice secreted by the human pancreas, as well as many other species, such as the cat, pig, and guinea-pig [113, 118]. Indeed, computer modelling of pancreatic HCO_3_^−^ secretion clearly showed that this arrangement of transporters would only be capable of achieving HCO_3_^-^ levels of ~80 mM [134, 135]. Partial resolution to this apparent problem came when the molecular identity of the putative Cl^−^/HCO_3_^−^ exchanger was identified and found to be due to expression of several members of the SLC26 family of anion transporters, SLC26A3 and SLC26A6 [136–138], which were introduced in the section on the airways. Both these exchangers are electrogenic with A3 transporting 2Cl^−^ for 1HCO_3_^−^ (therefore causing net Cl^−^ accumulation per transport cycle), while A6 transported 2HCO_3_^−^ out of the cell for one Cl^−^ into the cell [139]. Therefore, the direction and magnitude of anion transport is not only dependent on the prevailing chemical gradients for Cl^−^ and HCO_3_^−^ across the apical membrane, but is also affected by membrane potential. However, it is generally believed that SLC26A6 mediates the majority of the Cl^−^/HCO_3_^−^ exchange at the apical membrane based on inhibitor profile (DIDS-sensitivity) and studies from transgenic slc26a6 knock-out mice [140]. Furthermore, pioneering studies from the Muallem lab showed that both SLC26A exchangers were regulated by CFTR [141–144] through a physical interaction between the phosphorylated R domain of CFTR and the highly conserved STAS domain of the SLC26 transporter. This physical coupling between CFTR and the exchanger not only functionally activated anion exchange activity, but also had a positive effect on CFTR, and led to an increase in Po [136, 143], although the mechanism for both these effects is currently not understood. In addition, the physical coupling of these two anion transporters was shown to be facilitated by their binding to several scaffold proteins such as NHERF1 and CAP50, via their respective C-terminal PDZ binding motifs, which brings CFTR and SLC26A exchangers in close proximity, essentially forming a multimeric, anion transporting, complex [136, 141, 145]. In relation to HCO_3_^−^ secretion, and particularly in the case of SLC26A6, computer modelling showed that having a 2:1 electrogenic Cl^−^/HCO_3_^−^ transporter on the apical membrane, working with CFTR, could theoretically increase HCO_3_^−^ to ~120 mM [135]. Although this value was significantly higher than could be achieved by a 1:1 Cl^−^/HCO_3_^−^ exchanger, it was still less than the measured values in human pancreatic juice (140 mM). The reason for this was that the capacity of the exchanger to increase HCO_3_^−^ was found to be limited once luminal HCO_3_^−^ levels increased above 120 mM, since it would be operating close to equilibrium (i.e., very slowly), during the secretion of 140 mM HCO_3_^−^. Taken together with the observation that guinea-pig ducts can still secrete HCO_3_^−^ into a luminal fluid nominally free of Cl^−^ [146], this suggested that neither of the SLC26 transporters provided the main route for HCO_3_^−^ efflux across the apical membrane during *maximal* secretion. The final answer to the puzzle came from several observations. First, it was found that stimulation of HCO_3_^−^ secretion by cAMP agonists in intact guinea-pig ducts led to a very marked drop in intracellular Cl^−^ [147], and this reduction in intracellular [Cl^−^] was subsequently shown to activate a kinase cascade involving the WNK1-OSR1/SPAK pathway, that ultimately led to phosphorylation of CFTR (or a regulatory protein) and a marked increase in the relative permeability of CFTR to HCO_3_^−^ [148]. In addition, the low Cl^−^ concentration in the lumen also caused CFTR to shift its selectivity in favour of HCO_3_^−^ ions [149]. All of these factors, therefore, ensure that a HCO_3_^−^-rich secretion is produced with much of the secreted HCO_3_^−^ entering the lumen via CFTR.

This increase in the relative HCO_3_^−^ conductance of CFTR, together with the observed membrane potential of ~60 mV of stimulated duct cells [146, 150], was found to be capable of further increasing ductal HCO_3_^−^ levels to ~140–160 mM. However, when comparing ductal secretion from different species, it was also apparent that those species that produced a relatively low HCO_3_^−^-containing secretion (rat, mouse, and rabbit), had significant levels of NKCC1 activity, while high HCO_3_^−^ secretors did not, and instead expressed mainly the Na^+^-dependent HCO_3_^−^ transporter, NBCe1 (SLC4A4) [151], an electrogenic HCO_3_^−^ importer with a 2HCO_3_^−^ to 1Na^+^ stoichiometry [113, 152]. In the latter case, the NBC not only helped accumulate HCO_3_^−^ within the cell, by utilising the inward-directed sodium gradient across the basolateral membrane, but it also importantly helped maintain an electrical driving force for efflux of HCO_3_^−^ across the apical membrane, by offsetting the depolarisation of the cell due to electrogenic anion secretion [151, 153, 154]. The NBC, therefore, works in concert with potassium channels that are found in ductal cells, to maintain a negative membrane potential [155]. Furthermore, like CFTR, NBC activity is positively regulated by cAMP/PKA phosphorylation [156], and both proteins are synergistically regulated in a coordinated fashion by intracellular IRBIT, in a cAMP and calcium-dependent manner [157, 158]. Together, these data suggest that the flow-dependent changes in luminal Cl^−^/HCO_3_^−^ concentrations observed both in vivo and in vitro studies [113, 118] can be attributed to dynamic alterations in the mechanism of anion secretion through regulatory changes in CFTR and SLC26A6 activities. However, because HCO_3_^−^ secretion is controlled by the activity of CFTR, the production of a high luminal HCO_3_^−^ fluid must occur relatively high up in the ductal tree (intercalated/intralobular ducts), where CFTR is expressed [114, 115].

It should also be noted that there are inhibitory pathways that limit the extent of pancreatic HCO_3_^−^ and fluid secretion, probably to prevent excessive hydrostatic pressure building up within the ductal tree at high secretory flow rates [113]. Several studies have shown that these inhibitory pathways work through the release of neurotransmitters such as Substance P, that lead to a reduction in HCO_3_^−^ secretion by selectively reducing apical Cl^−^/HCO_3_^−^ exchange activity through a PKC-dependent pathway [159–161].

### Defects in CFTR-dependent HCO_3_^−^ secretion predispose to pancreatic disease

Several studies have indicated that insufficient ductal HCO_3_^−^ and fluid secretion leads to the destruction of the gland, as observed in the inherited disease CF [116, 162, 163]. An important consequence of impaired HCO_3_^−^ secretion is an acidic pancreatic juice (less than 6.5) that increases mucus viscosity, and decreases the solubility of secreted digestive enzymes. Both these factors predispose to the formation of mucin/protein plugs and eventually cysts within the ductal tree, as well as premature activation of digestive enzymes. This ultimately leads to the destruction of the gland which is one of the characteristic pathological features of CF of the pancreas [65]. As discussed in Chapter “Cystic Fibrosis: a clinical view”, mutations in the CFTR gene cause CF. There are over 2000 disease causing mutations which have been grouped into 5–6 classes based on the functional consequences of the mutation. Classes 1–3 are severe mutations, while Classes 4–5 are mild mutations. In relation to pancreatic pathology, ~85 % of people with CF are born pancreatic insufficient (PI), which equates to a reduction in pancreatic function of more than 95 %. In these people, there is a very good correlation between disease severity and the class of mutation [164–166] with ‘severe’ CF mutations, such as the most common CF mutation, F508del and the class 3 gating mutant, G511D, strongly correlating with PI. For those with ‘milder’ mutations (some residual channel activity such as Class 4, R117H), pancreatic function is preserved (pancreatic sufficient, PS), albeit to differing extents. However, in general, these PS individuals require less enzyme supplements, but can become PI with age. As described above, the increase in activity of the apical SLC26A6 anion exchanger during secretion appears to be strongly dependent on its interaction with CFTR, and interestingly, the exchanger is activated by a number of CFTR mutants that lack Cl^−^ channel activity [167]. This correlates with a good retention of pancreatic function in patients carrying those mutations [168]. Furthermore, anion transport studies from polarised cultures of the human CF pancreatic ductal cell line CFPAC, which is homozygous for F508del, showed that in addition to a lack of CFTR, apical SLC26A Cl^−^/HCO_3_^−^ exchange activity was also absent, despite evidence for mRNA expression. Importantly, anion exchange activity was restored upon viral-mediated CFTR transduction of the CFPAC cells [169]. Taken together, these results strongly suggest that a functional CFTR at the apical plasma membrane is a prerequisite for SLC26A-mediated anion exchange, and that mild CFTR mutations are likely to preserve Cl^−^/HCO_3_^−^ exchange activity, although this needs more formal demonstration.

Several recent investigations have also shown that two agents that classically induce stress/inflammation of the exocrine pancreas (pancreatitis), bile and alcohol, caused marked changes in HCO_3_^−^ and fluid secretion based on in vitro intracellular pH and fluid transport studies from isolated microdissected ducts. At low concentrations, both agents increased HCO_3_^−^ secretion, a response that required CFTR and Cl^−^/HCO_3_^−^ exchange activity [170–172]. However, higher levels of these agents led to a severe inhibition of CFTR-dependent HCO_3_^−^ secretion, which was due to profound mitochondrial damage and a consequent reduction in intracellular ATP levels [173, 174]. These studies were the first to suggest that ductal HCO_3_^−^ secretion could play a protective role against these noxious agents not hitherto thought of. Further support for this hypothesis came from studying the extent of pancreatic inflammation and necrosis caused by these agents in vivo, using either CFTR knock-out mice or a mouse model with reduced CFTR expression (NHERF1 KO mouse) [182]. In both cases, the extent of pancreatic pathology induced by administration of these noxious agents was significantly increased, highlighting a key role of CFTR in pancreatic protection. Furthermore, patients with autoimmune [175] or acute and chronic alcohol-induced pancreatitis, showed marked abnormalities in membrane localisation and expression levels of CFTR [176]. Finally, there is a significant correlation between the development of pancreatitis and variants in the CFTR gene that do not cause a typical CF phenotype, but appear to have impaired HCO_3_^−^ secretion [177–179].

In summary, CFTR plays an essential role in HCO_3_^−^ and fluid secretion in the exocrine pancreas. It regulates HCO_3_^−^ secretion in two fundamentally different ways; first, as a regulator of SLC26A-mediated Cl^−^/HCO_3_^−^ exchange, and second, as a direct exit pathway for HCO_3_^−^ secretion. Defects in CFTR-mediated HCO_3_^−^ transport lead to severe pancreatic dysfunction. Strategies for improving HCO_3_^−^ secretion in the CF pancreas are limited because of the marked tissue destruction at birth in the majority of people with CF. However, preliminary results from measurements of pancreatic function in young children with CF taking the Class 3 CFTR potentiator, Ivacaftor (see Chapter “Cystic Fibrosis: a clinical view”) over 24 weeks, have shown a significant restoration of enzyme-secreting capacity (increased faecal elastase-1 levels), and by inference, pancreatic tissue regeneration, which is an extremely exciting finding [180] that warrants further research. Furthermore, it is known that variants (SNPs) in the SLC26A9 anion transporter can influence disease severity in the CF lungs and gut (meconium ileus) and, therefore, act as gene modifiers. Importantly, a recent study has suggested that SNPs in SLC26A9 can also influence the degree of pancreatic insufficiency [181]. This opens up the possibility of targeting this anion transporter as a potential therapeutic target to slow the progression of exocrine dysfunction in CF (in addition to the lungs and ileum).

In terms of pancreatitis, recent animal studies have suggested that strategies that help maintain levels of HCO_3_^−^ secretion would limit the extent of pathology induced by bile and alcohol [174, 176, 182]. Furthermore, the effects of ethanol and ethanol metabolites on CFTR are consistent with reduced biogenesis, accelerated plasma membrane turnover, as well as channel inhibition [176]. Thus, restoring cell surface expression and activity of CFTR may partly alleviate the ethanol-induced damage. This potentially could be through the use of the FDA approved drug, Lumacaftor (see Chapter “Cystic Fibrosis: a clinical view”), which improves folding and processing of F508del-CFTR to the plasma membrane, as well as Ivacaftor to improve channel activity. Another goal here would be to find ways of preventing the marked reduction in ATP levels in ductal (and acinar) cells [125, 183]. A potential clinical strategy would be to try and improve nutritional support at a very early stage in acute pancreatitis, although a recent trial looking at the benefits of nasoenteric feeding after ~20 h of admission did not show any improvement in outcome compared to those patients that had on-demand oral feeding commencing at 24 h [184].

## Role of CFTR in sweat gland physiology

The human eccrine (or atrichal) sweat gland helps to maintain whole body temperature via the production of sweat in response to a hot environment, exercise, or emotional situations. An individual can secrete up to 4L of sweat in an hour to thermoregulate [185, 186]. Sweat consists primarily of water and salt, mostly NaCl but is hypotonic with respect to the interstitium. The sweat gets secreted onto the surface of the skin, where heat is lost from the body by the latent heat of evaporation of the sweat fluid. The ability to lose heat is affected by the prevailing outside temperature as well as humidity. However, it is increasingly being recognised that sweat has a number of other important roles which include the production and secretion of a range of AMPs such as LL-37, lactoferrin and dermicidin, an AMP unique to skin [187–189], as well as compounds that maintain skin condition (barrier function) and skin lubricants [190]. Sweat glands also contain stem cells important for renewing skin after wounding and burns [186, 191]. A number of disorders of sweat glands exist, with most involving defects in electrolyte and fluid production. These include CF, where lack of functional CFTR prevents normal NaCl absorption and leads to excessive salt loss ([192]; reviewed by [193]) which is discussed in more detail below; idiopathic anhidrosis (decreased volume of secretion), which may be caused by defects in calcium signalling [194, 195] and hyperhidrosis (uncontrolled and excessive sweat secretion), that may be due to changes in calcium signalling and water transport by aquaporin 5 [196, 197]). In addition, lack of sufficient antimicrobials can lead to skin infections and atopic dermatitis [198], and problems with wound healing and re-epithelialisation may be linked to stem cell dysfunction [191, 199, 200].

**Fig. 6:**
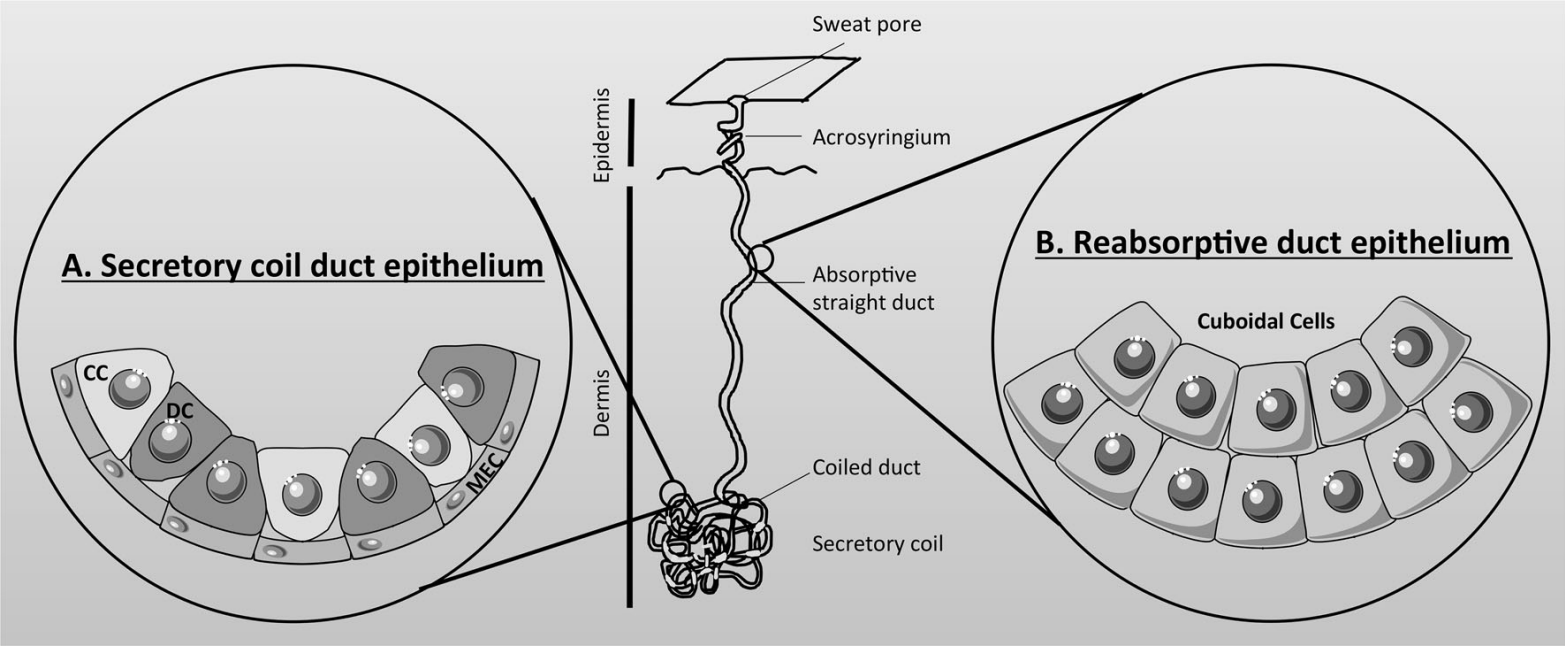
Schematic representation of an eccrine sweat gland. The human sweat gland is a simple-coiled tubular exocrine gland that resides in the dermis and connects to the surface of the skin by a straight absorptive duct. **a** The secretory coil duct epithelium is composed of clear (CC), dark (DC), and myoepithelial cells (MEC), and is responsible for producing the primary secretion. **b** The reabsorptive duct epithelium is composed of two layers of cuboidal cells which absorb salt but not water

Eccrine sweat glands are derived from embryonic ectoderm (as is the exocrine pancreas), and it is estimated that there are 2–4 million glands dispersed over the surface of the body, with the highest density on the forehead, palms of the hands and soles of the feet. The human sweat gland is a simple, coiled tubular, exocrine gland, 2–5 mm in length that resides in the lower part of the dermis, and which connects to the surface of the skin by a straight absorptive duct [186, 193]. The gland is composed of two structurally and functionally different units [201, 202] (Fig. 6). (1) The secretory coil (Fig. 6a) that makes up most of the coiled part of the gland and which is responsible for producing the primary secretion mainly in response to sympathetic cholinergic innervation, but beta-adrenergic stimulation also elicits a low volume secretion. The secretory coil is composed of three cell types. There is a single layer of epithelial cells, which consist of two morphologically and functional distinct types of cells known as clear (agranular) and dark (granular) cells which occur in equal proportions (Fig. 6a). These cells are responsible for producing the primary ‘sweat’ secretion, together with various glycoproteins and AMPs, respectively [185, 203]. Clear cells express CFTR [203, 204] as well as a CaCC, most probably TMEM16A (ANO1) [205]. The myoepithelial cells are the third type of cells (Fig. 6a), and are modified smooth muscle cells that contract during sweat secretion, and which provide structurally integrity to the secretory coil preventing damage to the tubule during gland stimulation [206]. (2) The absorptive straight duct (Fig. 6b) that acts to absorb NaCl, but not water, producing the final, hypotonic, sweat secretion that flows out to the skin. The absorption of salt, but not water, helps minimise salt loss from the body, which, if not controlled, could lead to circulatory collapse. Structurally, the proximal part of the absorptive duct is composed of a double layer of epithelial cells, which are electrically connected by gap junctions, and physically joined by desmosomes, to form a syncytium [202]. Further up towards the skin exit, the distal part of the duct consists of multiple layers of cells and is not thought to be involved in electrolyte or fluid transport [185].

### Role of CFTR in sweat gland electrolyte and fluid transport

CFTR is expressed in both portions of the sweat gland and has been shown to be involved in the elaboration of the primary secretion from the secretory coil in response to beta-adrenergic stimulation, as well as in the absorption of NaCl in the absorptive duct [193]. However, in the secretory coil, the major physiological stimulus is acetylcholine (ACh), which is released from post-ganglionic sympathetic cholinergic fibres, and which stimulates copious fluid transport through a non-CFTR dependent pathway. Although not fully resolved, the most likely Cl^−^ exit pathway is via the TMEM16A Cl^−^ channel, which has been localised to secretory cells [205], and which is activated by increases in cytosolic calcium via cholinergic stimulation. However, recent studies suggest that dark cells may also contribute to sweat secretion, but employ a distinct anion channel known as best2 (see [186] for further discussion), which may have an additional role in sweat pH and fluid regulation. Furthermore, studies from isolated sweat glands from adult mouse foot pads have shown expression of the mRNA for the Chloride Intracellular Channel 6 (Clic6), as well as for the slc26a4 Cl^−^/HCO_3_^−^ anion exchanger known as pendrin. The latter protein could play a role in HCO_3_^−^ secretion [207], as discussed in the section on the airways. In the clear cells at least, the production of the primary secretion involves accumulation of Cl^−^ above electrochemical equilibrium by a basolateral NKCC1, followed by Cl^−^ exit through apical-located Cl^−^ channels, CFTR or TMEM16A. This creates a lumen negative potential difference that drives paracellular transport of Na^+^, and water follows osmotically via aquaporins to produce an isotonic secretion, containing ~145 mM Na^+^, 115 mM Cl^−^, with the remaining anions being lactate and HCO_3_^−^ [185].

**Fig. 7:**
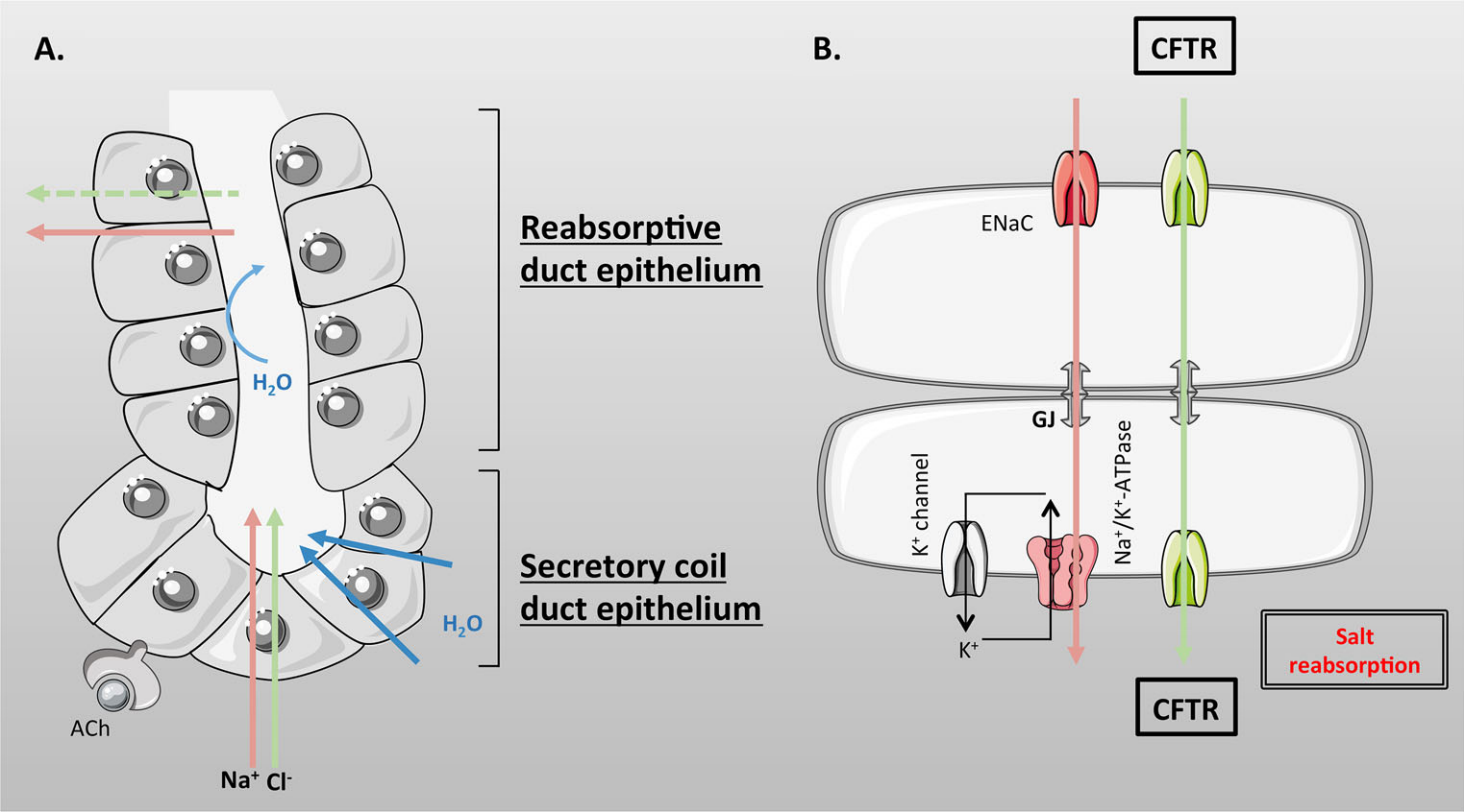
Ion transport in the sweat gland. **a** Secretory coil duct epithelial cells secrete NaCl and water mainly in response to cholinergic (acetylcholine, ACh) stimulation. **b** The reabsorptive duct epithelial cells express a constitutively active CFTR on both apical and basolateral membranes. Both Na^+^ and Cl^−^ move transcellularly and both ENaC and CFTR work together to regulate net transepithelial NaCl absorption. Na^+^ is pumped out of the cell across the basolateral membrane to the interstitial fluid by the Na^+^/K^+^-pump, generating a transepithelial electrical gradient favouring Cl^−^ absorption

The duct cells express the highest levels of CFTR, and in contrast to all other CF-affected epithelial tissues, the channel is functionally present at both apical and basolateral membranes [193, 208], and appears to be constitutively active [209] via PKA activity. Similar to the airways, ENaC is also expressed with CFTR at the apical membrane. However, there are marked differences in the way the two tissues operate (Compare Figs. 3 and 7). In the sweat duct, both Na^+^ and Cl^−^ move transcellularly and both channels work together to regulate net transepithelial NaCl absorption. Indeed, pioneering work from the Quinton lab showed that ENaC activity was dependent on a functional interaction with a phosphorylated, Cl^−^ transporting, CFTR [193, 210, 211], highlighting a completely different regulatory interaction between the two channels. Exactly how the activities of CFTR and ENaC are coordinated is still not fully established but may be due to changes in intracellular pH that accompany Na^+^ absorption through ENaC [212]. In the duct cells, (as in the airways) Na^+^ entry occurs through ENaC down a large electrochemical gradient. Intracellular Na^+^ is then pumped out of the cell across the basolateral membrane to the interstitial fluid by the Na^+^/K^+^-ATPase, generating a transepithelial electrical gradient favouring Cl^−^ absorption (Fig. 7b). However, unlike the airways, the paracellular pathway in the absorptive duct has little intrinsic Cl^−^ permeability in both normal and CF glands [208, 213] and due to high anion conductance of the apical and basolateral membranes of the duct cells (due to active CFTR), Cl^−^ moves transcellularly. Indeed, the declining Na^+^ concentration in the sweat fluid creates a driving force for passive Cl^−^ entry into the cell through active CFTR, and then, Cl^−^ exits via basolateral CFTR [185, 193]. Through this process NaCl concentration can fall to ~50 mM, before diffusion of Cl^−^ becomes limited by luminal Cl^−^ concentration [214], at least under normal flow rates. However, it has been observed that at low flow rates Cl^−^ levels can fall as low as 10–15 mM, and pH becomes very acidic [215], which must involve a different set of transporters. As discussed by Bovell [185], this could be due to a yet unidentified apical Cl^−^/HCO_3_^−^ exchanger that is coupled to proton-secretion via a V-type ATPase [216, 217], leading to net Cl^−^ absorption. Because of the low water permeability of the duct epithelium, NaCl absorption does not lead to concurrent water movement, and therefore, the resulting modified fluid is hypotonic, thus conserving valuable salt for whole body salt and fluid homeostasis [193].

### Sweat gland and cystic fibrosis

As discussed above, CFTR has an important role in electrolyte and fluid secretion as well as absorption. In people with CF, dysfunctional CFTR affects both processes, and this leads to two important clinical manifestations of the disease. (1) Although rates of cholinergic sweat secretion are very similar in non-CF and CF individuals, beta-adrenergic (cAMP) secretion is absent in CF [218]. (2) The inability of the sweat duct to absorb Cl^−^ prevents Na^+^ transport, and therefore, salt absorption is markedly reduced and sweat NaCl levels rise, producing an abnormally ‘salty’ sweat, one of the hallmarks of the disease. Although, under normal conditions, this is not a major clinical problem, during hot and humid conditions, people with CF can lose excessive salt and fluid, causing dehydration and heat prostration as originally observed by Dorothy Anderson [219]. Indeed, the increase in sweat forms the basis of the Quantitative Pilocarpine Iontophoresis Test (QPIT) that is still used today for diagnostic purposes [220, 221]. This test measures the concentration of Cl^−^ in sweat and requires iontophoretic introduction of pilocarpine (a cholinergic agonist) to stimulate local sweating and then sufficient sweat is collected for analysis of Cl^−^. Concentrations of Cl^−^ in excess of 60 mM in children are diagnostic of CF. Normal values are less than ~40 mM. More recently, four new assays have been introduced to assess both the secretory and reabsorptive capacity of sweat glands. These assays can measure changes in bioelectric potentials, which are higher in CF glands compared to normal [222], as well as skin impedance and rates of secretion, thus improving overall sensitivity and diagnostic value in sweat gland pathologies (see [221]) for a recent summary of these new tests).

In summary, CFTR plays an important role in both salt and fluid secretion and absorption in the sweat gland, which is similar to the airways. However, in marked contrast to the airways, the ability of CFTR to regulate NaCl absorption relies on a positive interaction and regulation of ENaC; in other words, CFTR works with ENaC, and not against it! Exactly how two channels in the same membrane have completely different regulatory interactions is intriguing but poorly understood, and requires further research. Uniquely, CFTR is expressed in both apical and basolateral membranes of duct epithelial cells, although the underlying mechanism for this dual targeting has not been elucidated. Undoubtedly, cell/tissue specific regulatory interactions will have a role to play, but little is known in the sweat gland of these processes. It is intriguing that recent work form the CF pig has shown CFTR is not expressed in the plasma membrane of smooth muscle cells but is targeted to the sarcoplasmic reticulum [223], which illustrates the importance of cell context in relation to CFTR targeting. Defects in CFTR-mediated Cl^−^ transport lead to severe sweat gland dysfunction and excessive salt loss in CF. Although not a major clinical problem, there is no doubt that the sweat gland has been pivotal to our general understanding of the role of CFTR in CF which has been eloquently summarised in a recent review by Paul Quinton [193], and it still holds great promise as a diagnostic tool for evaluation of new therapies for CF in this age of personalised (*N* = 1) medicine [224].

## Final summary

CFTR has a central role in coordinating electrolyte and fluid transport in a range of epithelial tissues including the airways, GI and reproductive tracts and secretory glands. It achieves this not only by acting as a conduit for anion transport (its ion channel function), but also through its varied and complex regulation of other, non-CFTR channels, and anion transporters (its transporter regulatory function), that also participate in salt and fluid transport. Through this activity, CFTR has a key role in maintaining epithelial integrity and defence of the body as a whole. Although we have only described the role of CFTR in three epithelial tissues, we chose these primarily to illustrate the varied functional roles of CFTR. Understanding the basis for these different activities of CFTR has not only helped us gain a better understanding of the role of CFTR in the physiology of the tissue, but importantly, has led to the development of new and better strategies to help overcome CFTR-related pathologies. This is best exemplified by the development of a new generation of small molecule therapies being used to treat the basic defect in CF, which is described in more detail in Chapter “Cystic Fibrosis: a clinical view”. However, this approach is also being applied to CFTR-dependent secretory diarrhoeas, and may be relevant to other disease in which a link to CFTR malfunction is emerging, such as acute pancreatitis, COPD and asthma. However, we still lack precise details about the underlying molecular mechanisms that endow CFTR with the ability to modulate other transporters and defence mechanisms, so further research is clearly important and warranted.
